# Del Nido *vs.* Cold Blood Cardioplegia for High-Risk Isolated Coronary Artery Bypass Grafting in Patients with Reduced Ventricular Function

**DOI:** 10.21470/1678-9741-2022-0346

**Published:** 2024-01-30

**Authors:** Krzysztof Sanetra, Witold Gerber, Marta Mazur, Marta Kubaszewska, Ewa Pietrzyk, Piotr Paweł Buszman, Paweł Kaźmierczak, Andrzej Bochenek

**Affiliations:** 1 Division of Cardiovascular Surgery, Andrzej Frycz Modrzewski Krakow University, Krakow, Poland; 2 Department of Cardiac Surgery, American Heart of Poland, Bielsko-Biała, Poland; 3 Department of Cardiac Surgery, Academy of Silesia, Katowice, Poland; 4 Department of Cardiology, Andrzej Frycz Modrzewski Krakow University, Krakow, Poland; 5 Center for Cardiovascular Research and Development, American Heart of Poland, Katowice, Poland; 6 Department of Cardiology, American Heart of Poland, Bielsko-Biała, Poland; 7 American Heart of Poland, Katowice, Poland

**Keywords:** Cardiopulmonary Bypass, Myocardial Infarctation, Extracorporeal Circulation, Creatine Kinase, Biomarkers

## Abstract

**Introduction:**

The evidence for using del Nido cardioplegia protocol in high-risk patients with reduced ejection fraction undergoing isolated coronary surgery is insufficient.

**Methods:**

The institutional database was searched for isolated coronary bypass procedures. Patients with ejection fraction < 40% were selected. Propensity matching (age, sex, infarction, number of grafts) was used to pair del Nido (Group 1) and cold blood (Group 2) cardioplegia patients. Investigation of biomarker release, changes in ejection fraction, mortality, stroke, perioperative myocardial infarction, composite endpoint (major adverse cardiac and cerebrovascular events), and other perioperative parameters was performed.

**Results:**

Matching allowed the selection of 45 patient pairs. No differences were noted at baseline. After cross-clamp release, spontaneous sinus rhythm return was observed more frequently in Group 1 (80% vs. 48.9%; P=0.003). Troponin values were similar in both groups 12 and 36 hours after surgery, as well as creatine kinase at 12 hours. A trend favored Group 1 in creatine kinase release at 36 hours (median 4.9; interquartile range 3.8-9.6 ng/mL vs. 7.3; 4.5-17.5 ng/mL; P=0.085). Perioperative mortality, rates of myocardial infarction, stroke, or major adverse cardiac and cerebrovascular events were similar. No difference in postoperative ejection fraction was noted (median 35.0%; interquartile range 32.0-38.0% vs. 35.0%; 32.0-40.0%; P=0.381). There was a trend for lower atrial fibrillation rate in Group 1 (6.7% vs. 17.8%; P=0.051).

**Conclusion:**

The findings indicate that del Nido cardioplegia provides satisfactory protection in patients with reduced ejection fraction undergoing coronary bypass surgery. Further prospective trials are required.

## INTRODUCTION

**Table:** 

Abbreviations, Acronyms & Symbols	
ACT	= Activated clotting time
AF	= Atrial fibrillation
AKI	= Acute kidney injury
AKIN	= Acute Kidney Injury Network
CB	= Cold blood cardioplegia group
CK-MB	= Creatine kinase (MB isoenzyme)
COPD	= Chronic obstructive pulmonary disease
DN	= del Nido cardioplegia group
ECC	= Extracorporeal circulation
EF	= Ejection fraction
EuroSCORE	= European System for Cardiac Operative Risk Evaluation
hsTnT	= High-sensitivity troponin T
IABP	= Intra-aortic balloon pump
LIMA	= Left internal mammary artery
MACCE	= Major adverse cardiac and cerebrovascular events
MI	= Myocardial infarction
PCI	= Percutaneous coronary intervention

The cold blood cardioplegia concept was originally designed by Follette et al.^[1]^, and since then, it has had a well-established role in adult cardiac surgery. Its use in high-risk surgical procedures is justified both theoretically and practically. Using blood as a vehicle improves oxygen delivery, ensures a high buffering capacity, improves capillary distribution, delivers autologous free radical scavengers, provides adequate oncotic pressure, and maintains haemodilution in a safe, physiological range. These features may play a key role in patients with depressed myocardial function undergoing coronary surgery, as they are expected to tolerate any additional ischemic injury poorly.

On the other hand, the interest in using del Nido cardioplegia has been growing in recent years. Adequate myocardial protection comparable to blood cardioplegia has already been proven in randomized clinical trials^[2-4]^. Although single-dose cardioplegia delivery was already evaluated in coronary artery bypass grafting procedures, the evidence regarding using this solution in cases with severely impaired systolic ventricular function is limited.

However, there is no clear evidence comparing both solutions in high-risk patients with severely impaired myocardial contractility undergoing isolated coronary surgery. To address this issue, a retrospective analysis was performed to evaluate the efficacy of del Nido cardioplegia (Group 1) and cold blood cardioplegia (Group 2) in patients with significantly reduced ejection fraction (EF) who underwent isolated coronary artery bypass grafting.

## METHODS

### Patients

An anonymized database was created from the hospital registry (from 2014 to 2021) to search for patients who underwent isolated coronary artery bypass grafting procedures. Further selection criteria included preoperative EF < 40% (heart failure with reduced EF) and procedural use of either cold blood or del Nido cardioplegia. Patients who required additional procedures or had retrograde cardioplegia delivery were excluded from the study.

### Research Ethics Board Consent

This study is a retrospective analysis of a fully anonymized institutional dataset. No further interventions were made to subjects, and all data were readily available. As such, following the National Code for Clinical Trials (National Code on Clinical Researches, 2011), research ethics board consent is not mandatory for the quantitative part of the study. Furthermore, all admitted patients consented to medical data processing for scientific purposes before admission to the hospital.

### Surgical Treatment of Coronary Artery Disease - Qualification and Strategy

Before the procedure, coronary angiography was carefully evaluated. The qualification process throughout the entire period was based on the actual European Society for Cardiology/European Association for Cardiothoracic Surgery Clinical Guidelines^[5,6]^. Our institution has a Heart Center structure, so the heart-team assessment was performed whenever indicated.

Our facility routinely uses the left internal mammary artery (LIMA) for left anterior descending grafting. Other coronary arteries are revascularized using the saphenous vein. However, in patients younger than 50 years old, total arterial revascularization is the preferred method.

### Anesthesia and Preparation for Coronary Artery Bypass Grafting

Combined general anesthesia was used for every patient. Each patient was intubated, and a central venous port was introduced, preferably via the right jugular vein. A Foley catheter was placed into the bladder. At the end of LIMA harvesting, unfractionated heparin was administered at a calculated dose adjusted to the patient’s body mass to achieve an activated clotting time (ACT) of at least 480 seconds. After completing the procedure, anticoagulation was reversed with protamine sulfate (1.5 mg/100 units of heparin).

### Surgical Procedure

Median sternotomy was used for all cases. LIMA was harvested using low-energy coagulation. The bridging harvesting technique was used for saphenous vein harvest, and the standard surgical technique was used for radial artery harvest. After achieving an adequate ACT, an arterial cannula was inserted into the ascending aorta, and a double-staged venous cannula was inserted into the right atrium. Cardiopulmonary bypass was initiated. After placing the cross-clamp and administering cardioplegia (following the specific protocol below), distal anastomoses were performed using 7.0 or 8.0 sutures. The cross-clamp was released, and the return of rhythm was observed. Defibrillation was performed whenever necessary. Proximal anastomoses were performed on the ascending aorta using 6.0 sutures with the vessel partially clamped. Subsequently, weaning from extracorporeal circulation was done, and the cannulas were removed. After reversing anticoagulation, chest tubes were inserted into the left pleura and pericardium, and the sternum was closed with surgical wires. The subcutaneous tissue and skin were then sutured.

### Cardioplegia Protocol

Antegrade cardioplegia was administered to most of the patients at our institution. Retrograde cardioplegia was rarely used and reserved for selected cases. The cold blood cardioplegia used in our protocol contained 500 mL of crystalloid, consisting of 435 mL Plasma-Lyte A, 20 mL mannitol 15%, 20 mL NaHCO₃ 8.4%, and 25 mL KCl 2 mEq/mL. The crystalloid was mixed with autologous blood in a ratio of 4:1 (blood:crystalloid). The electrolyte concentration and administration protocol are provided in Table 1.

**Table 1:** Cardioprotection protocol comparison.

Components in crystalloid volume	Del Nido cardioplegia (1059 ml)	Cold blood cardioplegia (500 ml)
Na^+^ (mEq)	153	81
K^+^ (mEq)	31	52
Ca^++^ (mEq)	-	-
Mg^++^ (mEq)	19.24	1.3
HCO3^-^ (mEq)	13	20
Cl^-^ (mEq)	124	92.6
CH₃COO^-^ (mEq)	27	11.7
C₆H₁₁O₇^-^ (mEq)	23	10
Lidocaine (g)	0.130	-
Mannitol (g)	3.26	3
Blood additive	Yes; 1:4 (blood:crystalloid)	Yes; 4:1 (blood:crystalloid)
Dosage (mL)	20 mL/kg; maximal single dose: 1500 mL	15 mL/kg; additional 5 mL/kg every 20-30 minutes
Infusion pressure (mmHg)	100-200	100-200
Infusion flow (mL/min)	200-300	200-300
Infusion temperature (°C)	4-ago.	4-ago.

The crystalloid component of the del Nido cardioplegia was prepared using calculated doses to provide a solution identical to the originally described protocol (1000 mL Plasma-Lyte A, 16.3 mL mannitol 20%, 4 mL MgSO4 50%, 13 mL NaHCO₃ 8.4%, 13 mL KCl 2 mEq/mL, and 13 mL lidocaine 1%). The crystalloid was mixed with autologous blood in a ratio of 4:1 (crystalloid:blood). The electrolyte concentration and administration protocol are provided in Table 1. A dose of 20-40 mg furosemide was given unless intraoperative hemofiltration was used to manage high volume given in a single dose.

### Propensity Matching

To improve the precision of the study, propensity matching was performed. The matched parameters included age (maximum difference tolerance: three years), sex (male/female), the number of bypass grafts (exact number), and the presence of myocardial infarction at baseline (yes/no).

### Primary Endpoints

To assess the cardioprotective capability of the propensity-matched group of patients, biomarker release and EF were considered as primary measurements since they are routinely performed for all patients within the same time frame. High-sensitivity troponin T, with a reference range of 0-14 pg/mL, and creatine kinase-MB isoenzyme, with a reference range of 0-7 ng/mL, were measured 12 and 36 hours after surgery.

Transthoracic echocardiography and Simpson’s method were used to assess left ventricular systolic function preoperatively and on the second postoperative day.

### Other Measured Parameters

Baseline parameters, including planned surgery/acute coronary syndrome, age, sex, comorbidities, and perioperative risk in European System for Cardiac Operative Risk Evaluation (or EuroSCORE) II, were recorded for each patient. Major adverse cardiac and cerebrovascular events (MACCE) (defined as death, myocardial infarction, or stroke) was evaluated throughout the hospitalization. Myocardial infarction was defined following the fourth universal definition of myocardial infarction, which includes an elevation of troponin values > 10-times the 99^th^ percentile upper reference limit in patients with normal baseline troponin values or a rise > 20% in patients with elevated but stable troponin values, plus one of the following: the development of new pathological Q waves, angiographically documented new graft occlusion or new native coronary artery occlusion, imaging evidence of new loss of viable myocardium, or new regional wall motion abnormality consistent with an ischemic etiology^[7]^.

Our institution routinely uses inotropic support for patients with significantly impaired contractility when weaning from extracorporeal circulation. Therefore, mechanical circulatory support rates (mainly intra-aortic balloon pump [IABP]) were compared to assess the issue of low cardiac output despite the use of inotropes.

Daily creatinine values were measured, and the highest values were compared with preoperative creatinine to determine renal dysfunction following the Acute Kidney Injury Network criteria: stage 1 (creatinine increase > 0.3 mg/dL or 150-200%), stage 2 (creatinine increase 200-300%), and stage 3 (creatinine increase > 300%).

### Power Calculations

An independent samples *t*-test assuming unequal variances was used to estimate continuous data. A difference in means of 14 pg/mL troponin values and a standard deviation of 14 pg/mL would require 22 pairs at a 95% confidence level and 90% power to achieve statistical significance. A difference in creatine kinase means of 7 ng/mL and a standard deviation of 7 ng/mL would also require 22 pairs at a 95% confidence level and 90% power to achieve significance. For EF, a difference in means of 5% and a standard deviation of 5% would require 13 pairs at a 95% confidence level and 90% power for the results to become significant.

### Statistical Analysis

Continuous data are presented as median (interquartile range), while categorical data are presented as number (percentage). The Shapiro-Wilk test was used to assess the distribution of continuous data (normal *vs.* non-normal). Normal distribution was rejected for nearly all continuous parameters except cross-clamping and extracorporeal circulation time in Group 1. Therefore, the Mann-Whitney U test was used for comparative calculations. Fisher’s exact test was used to compare categorical data due to the small sample size. No missing values were reported for the analyzed endpoints. The alpha level was set at 0.05, and calculations were performed using MedCalc v.18.5 (MedCalc Software, Ostend, Belgium).

## RESULTS

There were 130 patients with reduced EF (< 40%) who underwent isolated coronary artery bypass grafting at our facility between 2014 and 2021, which accounted for 5.2% of the total number of 2,524 coronary artery bypass procedures that utilized extracorporeal circulation. The mortality rate in this group was 1.5% (two cases), and the occurrence of MACCE was 3.8% (five cases). Of this cohort, 76 patients received del Nido cardioplegia, while 54 received cold blood cardioplegia.

Propensity matching was used to include 45 pairs of patients in the analysis. At baseline, there were no differences in the patients’ characteristics, as they presented with similar occurrences of comorbidities and had equal preoperative biomarker values and EF (Table 1). Perioperative risk in both groups was also similar. Sixteen matched pairs (35.5%) received two bypass grafts, 27 pairs (60%) received three bypass grafts, and two pairs (4.4%) received four bypass grafts. The changes in the number and volume of cardioplegia administration reflected the protocol (Table 2). After the cross-clamp was released, spontaneous sinus rhythm return was more frequent in patients who received del Nido cardioplegia (Table 3).

**Table 2:** Baseline patients’ characteristics.

	Group 1	Group 2	*P*-value
	Del Nido cardioplegia (n = 45)	Cold blood cardioplegia (n = 45)	
Age (years)	66.0 (63.0 - 74.5)	69.0 (64.0 - 74.0)	0.282
Male sex	37 (82.2%)	37 (82.2%)	1
History of MI	15 (33.3%)	19 (42.2%)	0.514
1 MI	10 (22.2%)	15 (33.3%)	0.347
2 or more MI	5 (11.1%)	4 (8.9%)	1
MI on admission	6 (13.3%)	6 (13.3%)	1
History of PCI	10 (22.2%)	12 (26.6%)	0.807
Arterial hypertension	34 (75.5%)	33 (73.3%)	1
Diabetes	8 (17.8%)	12 (26.7%)	0.320
Hyperlipidemia	19 (42.2%)	22 (48.8%)	0.672
Obesity	5 (11.1%)	3 (6.7%)	0.714
Nicotinism	6 (13.3%)	3 (6.7%)	0.484
Hypothyroidism	1 (2.2%)	2 (4.4%)	1
Kidney disease	5 (11.1%)	6 (13.3%)	1
Asthma/COPD	1 (2.2%)	2 (4.4%)	1
Paroxysmal AF	1 (2.2%)	1 (2.2%)	1
Persistent/permanent AF	1 (2.2%)	1 (2.2%)	1
Preoperative EF	35.0 (30.0 - 38.0)	35.0 (29.5 - 35.5)	0.442
Preoperative hsTnT	20.6 (10.7 - 52.4)	25.8 (17.2 - 73.9)	0.111
Preoperative CK-MB	17.6 (11.3 - 25.9)	20.6 (9.3 - 49.3)	0.810
EuroSCORE II (%)	1.9 (1.1 - 2.6)	2.2 (1.5 - 3.1)	0.723

Data are presented as number (percentage) and median (interquartile range)

AF=atrial fibrillation; CK-MB=creatine kinase (MB isoenzyme); COPD=chronic obstructive pulmonary disease; EF=ejection fraction; EuroSCORE=European System for Cardiac Operative Risk Evaluation; hsTnT=high-sensitivity troponin T; MI=myocardial infarction; PCI=percutaneous coronary intervention

**Table 3:** Surgical procedure.

	Group 1	Group 2	*P*-value
	Del Nido cardioplegia (n = 45)	Cold blood cardioplegia (n = 45)	
Median graft number	3.0 (2.0 - 3.0)	3.0 (2.0 - 3.0)	1
Total arterial revascularization	1 (2.2%)	1 (2.2%)	1
1 cardioplegia infusion	45 (100%)	5 (11.1%)	< 0.001
2 cardioplegia infusions	0	34 (75.5%)	< 0.001
> 2 cardioplegia infusions	0	6 (13.3%)	< 0.001
Median infusion number	1.0	2.0	< 0.001
Intraoperative hemofiltration	4 (8.9%)	6 (13.3%)	0.739
Spontaneous sinus rhythm return	36 (80%)	22 (48.9%)	0.003
Cross-clamping time (min.)	48.5 (36.5 - 56.0)	53.0 (41.0 - 57.0)	0.249
ECC duration (min.)	64.0 (55.0 -75.0)	70.0 (58.0 - 80.0)	0.107

Data are presented as number (percentage) and median (interquartile range)

ECC=extracorporeal circulation

**Fig. 1:**
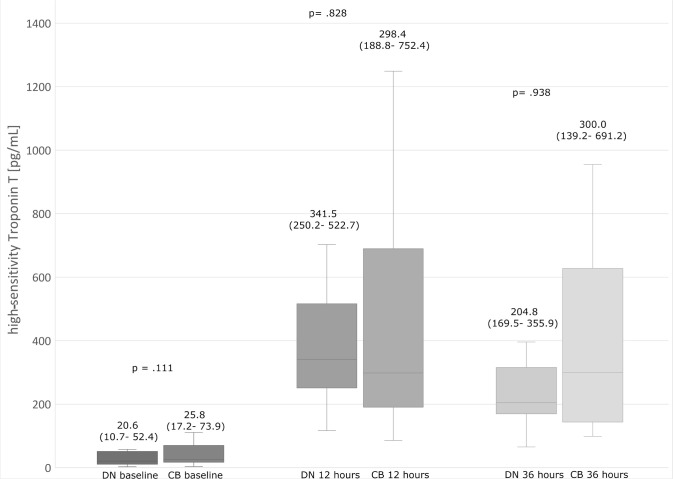
High-sensitivity troponin T values in time intervals. The upper and lower borders of the box represent the upper and lower quartiles. The middle horizontal line represents the median. The upper and lower whiskers represent the maximum and minimum values of nonoutliers. CB=cold blood cardioplegia group; DN=del Nido cardioplegia group.

**Fig. 2:**
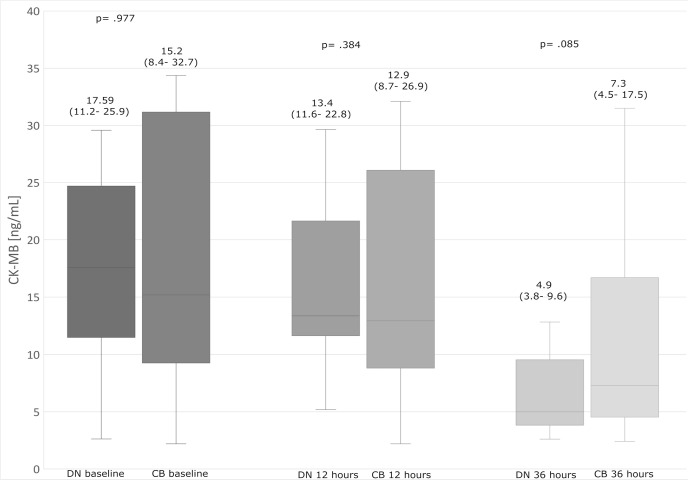
Creatine kinase (MB isoenzyme) (CK-MB) values in time intervals. The upper and lower borders of the box represent the upper and lower quartiles. The middle horizontal line represents the median. The upper and lower whiskers represent the maximum and minimum values of nonoutliers. CB=cold blood cardioplegia group; DN=del Nido cardioplegia group.

**Fig. 3:**
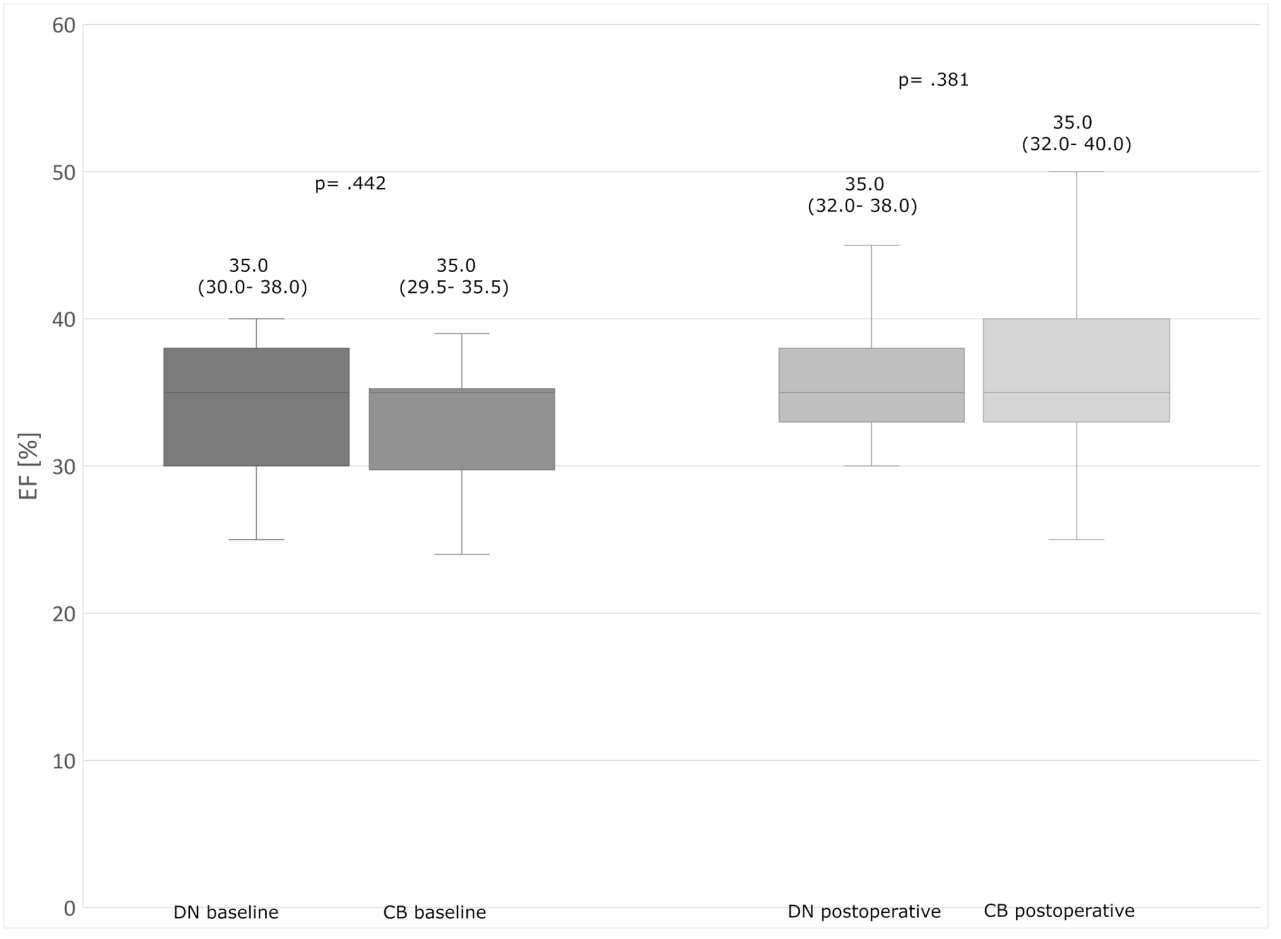
Ejection fraction (EF) values in time intervals. The upper and lower borders of the box represent the upper and lower quartiles. The middle horizontal line represents the median. The upper and lower whiskers represent the maximum and minimum values of nonoutliers. CB=cold blood cardioplegia group; DN=del Nido cardioplegia group.

The patients had insignificant differences in postoperative biomarker release (Figures 1 and 2) and postoperative EF (Figure 3). Mortality, postoperative myocardial infarction and stroke incidence, or the composite endpoint (MACCE) were similar in both groups (Table 4). There was a strong trend in favor of del Nido cardioplegia in the occurrence of new-onset postoperative atrial fibrillation (AF). There was also a trend for a lower incidence of kidney injury in Group 1, and the hospitalization time was shorter in Group 1.

**Table 4:** Perioperative analysis.

	Group 1	Group 2	*P*-value
	Del Nido cardioplegia (n = 45)	Cold blood cardioplegia (n = 45)	
Death (any cause)	0	1 (2.2%)	1
Myocardial infarction	0	2 (4.4%)	0.494
Perioperative IABP	1 (2.2%)	4 (8.9%)	0.361
Stroke	1 (2.2%)	0	1
MACCE	1 (2.2%)	2 (4.4%)	1
EF decrease > 5%	1	1	1
Chest revision	1 (2.2%)	2 (4.4%)	1
New-onset AF	3 (6.7%)	8 (17.8%)	0.051
Delirium	3 (6.7%)	2 (4.4%)	1
Transfusions	4 (8.9%)	3 (6.7%)	1
Acute kidney injury	7 (15.6%)	15 (33.3%)	0.085
AKIN 1 (150-200% baseline creatinine)	6 (13.3%)	11 (20.0%)	0.281
AKIN 2 (200-300% baseline creatinine)	1 (2.2%)	4 (8.9%)	0.361
Highest creatinine (mg/dl)	1.3 (1.2 - 1.7)	1.4 (1.2 - 1.9)	0.439
Hemodiafiltration	0	2	0.092
Hospitalization period (days)	6.0 (5.0 - 7.0)	7.0 (6.0 - 8.5)	0.005

Data are presented as number (percentage) and median (interquartile range)

AF=atrial fibrillation; AKIN=Acute Kidney Injury Network; EF=ejection fraction; IABP=intra-aortic balloon pump; MACCE=major adverse cardiac and cerebrovascular events

## DISCUSSION

Many surgeons have raised doubts regarding the use of del Nido cardioplegia for coronary artery bypass grafting. Apart from the advantageous effect of using blood as a vehicle, the single delivery, particularly with no simultaneous retrograde infusion, is believed to be inferior in terms of providing uniform myocardial protection in hearts with impaired coronary circulation.

Several studies have addressed these doubts, and their authors have concluded that, in most cases, del Nido cardioplegia may be used as a safe alternative for conventional cardioplegia in coronary surgery. It is associated with shorter cross-clamping times, a trend towards lower transfusion rates, a high number of spontaneous sinus rhythm returns, and even lower infection rates^[8-11]^. However, in a randomized trial comparing del Nido and blood cardioplegia in coronary surgery, no advantage of one over the other was proven[4].

However, none of the mentioned studies focused on patients with severely impaired systolic myocardial function. Referring to analyses of coronary artery bypass grafting patients with high-risk surgery, Yerebakan et al.^[10]^ gathered a post-myocardial infarction group with a median EF > 40% and reaching 49% in the del Nido cardioplegia group. The authors did not notice any differences in transfusion rates, length of stay, IABP requirement, postoperative inotropic support, and 30-day mortality, which is similar to our results.

On the other hand, Gunaydin et al.^[12]^ (who also presented groups with a mean EF > 40%) reported higher interleukin-6 and cardiac troponin levels in the del Nido group, along with more frequent AF attacks and hospital readmissions. This contradicts our results, and the matter surely requires further investigation.

The literature referring to the use of blood cardioplegia in high-risk cases is much more extensive. Recent experimental analyses indicate that blood cardioplegia may be superior to crystalloid cardioplegia in infarcted hearts, as it is associated with better preservation of myocardial function^[13]^. Furthermore, Flack et al.^[14]^ reported that blood cardioplegia and combined antegrade and retrograde cardioplegia are superior to crystalloid and antegrade cardioplegia for postoperative morbidity in patients with advanced left ventricular dysfunction, but the authors noted no significant difference in early or late survival. However, at this point, it must be noted that the del Nido protocol is not purely crystalloid, and the blood additive may significantly impact cardioprotection, which must be considered when addressing those studies.

Referring to secondary observations from our report, it must be noted that although no statistical significance was gained due to small groups, postoperative acute kidney injury (AKI) incidence was twice as frequent in patients who received cold blood cardioplegia. This matter requires further investigation in a prospective study since kidney injury after cardiac operations is one of the most common causes of AKI (second on the list, following sepsis). It is also correlated with both mortality and morbidity independently^[15]^. Sorabella et al.^[16]^ noted lower postoperative creatinine values in reoperative aortic valve patients in whom del Nido cardioplegia was used, but no significance was found. Furthermore, in a randomized trial comparing del Nido and blood cardioplegia in patients who underwent aortic valve surgery, statistical significance was reached for the superiority of del Nido cardioplegia in this matter^[3]^. However, the study was not designed for kidney injury analysis; multiple secondary endpoints existed. Consequently, the risk of type 1 error is too high to confirm the true significance of this finding. It must be noted that lidocaine is believed to have a nephroprotective effect due to vasodilatation and preventing messenger ribonucleic acid dysregulation^[17-18]^.

A similar prospective evaluation should be performed for AF. In our cohort, the arrhythmia was three times more frequent in patients who received cold blood cardioplegia, with a strong statistical trend but no significance (*P*=0.051). This finding is consistent with a recent study that revealed a lower incidence of postoperative AF in coronary artery bypass grafting procedures when del Nido cardioplegia was used^[19]^. It may be speculated that we would also reach statistical significance in a slightly greater number of patients. The potential difference in this matter has a great clinical consequence, as AF worsens the postoperative prognosis and increases the length of intensive care unit stay, hospitalization, and hospital costs^[20]^. The antiarrhythmic role of lidocaine may be hypothesized, which would also explain why we noted a higher incidence of spontaneous sinus rhythm return in the del Nido cardioplegia group (*P*=0.003). This last finding is similar to clinical trials and experimental studies^[3,21]^, but its clinical implications are yet to be investigated.

### Limitations

The study is a retrospective dataset analysis; in such cases, all issues associated with this methodology must be considered. Additionally, the endpoints only refer to the hospitalization period. Furthermore, the small sample size is one of the main limitations, indicating that further investigation is mandatory.

## CONCLUSION

The findings indicate that del Nido cardioplegia provides satisfactory protection in patients with reduced EF undergoing coronary bypass surgery and may be used as a safe alternative for blood cardioplegia. However, validating major findings in a prospective clinical trial is mandatory.

**Table:** 

Authors’ Roles & Responsibilities	
KS	Substantial contributions to the conception or design of the work; or the acquisition, analysis, or interpretation of data for the work; drafting the work or revising it critically for important intellectual content; agreement to be accountable for all aspects of the work in ensuring that questions related to the accuracy or integrity of any part of the work are appropriately investigated and resolved; final approval of the version to be published
WG	Substantial contributions to the conception or design of the work; or the acquisition, analysis, or interpretation of data for the work; drafting the work or revising it critically for important intellectual content; final approval of the version to be published
MM	Substantial contributions to the conception or design of the work; or the acquisition, analysis, or interpretation of data for the work; final approval of the version to be published
MK	Substantial contributions to the conception or design of the work; or the acquisition, analysis, or interpretation of data for the work; final approval of the version to be published
EP	Substantial contributions to the conception or design of the work; or the acquisition, analysis, or interpretation of data for the work; final approval of the version to be published
PPB	Substantial contributions to the conception or design of the work; or the acquisition, analysis, or interpretation of data for the work; agreement to be accountable for all aspects of the work in ensuring that questions related to the accuracy or integrity of any part of the work are appropriately investigated and resolved; final approval of the version to be published
PK	Substantial contributions to the conception or design of the work; or the acquisition, analysis, or interpretation of data for the work; final approval of the version to be published
AB	Substantial contributions to the conception or design of the work; or the acquisition, analysis, or interpretation of data for the work; drafting the work or revising it critically for important intellectual content; final approval of the version to be published
